# Tongue metastasis of cutaneous melanoma: Report of two cases and literature review 

**DOI:** 10.4317/jced.54980

**Published:** 2018-11-01

**Authors:** Renata-Lucena Markman, Giuliano-Augusto-Belizario Rosa, Leonardo Cardili, Luciana-Estevam Simonato, Thais-Bianca Brandão, Ana-Carolina-Prado Ribeiro

**Affiliations:** 1 DDS, MSc, PhD. Oral Diagnosis Department, Semiology Area, Piracicaba Dental School, University of Campinas (UNICAMP), Piracicaba, São Paulo, Brazil. Av. Limeira, 901, Areão, Piracicaba, São Paulo, Brazil; 2 DDS. Dental Oncology Service, Instituto do Câncer do Estado de São Paulo [ICESP], Faculdade de Medicina da Universidade de São Paulo, São Paulo, Brazil. Av. Dr. Arnaldo, 251, Cerqueira César, São Paulo, Brazil; 3 MD, MSc. Pathology Service, Instituto do Câncer do Estado de São Paulo [ICESP], Faculdade de Medicina da Universidade de São Paulo, São Paulo, Brazil. Av. Dr. Arnaldo, 251, Cerqueira César, São Paulo, Brazil; 4 DDS, MSc, PhD.Universidade Brasil, Campus Fernandópolis, São Paulo, Brazil. Est. Projetada F-1, s/n – Fazenda Santa Rita, Fernandópolis, São Paulo, Brazil; 5 DDS, MSc, PhD. Dental Oncology Service, Instituto do Câncer do Estado de São Paulo [ICESP], Faculdade de Medicina da Universidade de São Paulo, São Paulo, Brazil. Av. Dr. Arnaldo, 251, Cerqueira César, São Paulo, Brazil; 6 DDS, MSc, PhD. Dental Oncology Service, Instituto do Câncer do Estado de São Paulo [ICESP], Faculdade de Medicina da Universidade de São Paulo, São Paulo, Brazil. Av. Dr. Arnaldo, 251, Cerqueira César, São Paulo, Brazil

## Abstract

**Introduction:**

Malignant metastases to the oral cavity are rare and metastatic melanomas of the tongue are considered exceptionally uncommon, with less than 10 cases published in the English literature so far.

**Case reports:**

Two female patients in the 7th decade of life presented to our dental service with nodules in the tongue. Both patients had multiple metastases at the time of oral diagnosis and primary melanoma originated on the skin. An intra-oral incisional biopsy was performed under local anesthesia and the histopathologic analysis was characterized by the proliferation of atypical epithelioid cells displaying a poorly delimited cytoplasm and hyperchromatic nucleus which contained eosinophilic macronucleoli. Immunohistochemistry was performed in both cases to confirm the clinical hypothesis of metastatic melanoma. After the diagnosis of oral metastatic melanoma, the patients were maintained under palliative care and close medical follow-up. Both patients died four and a half months and 20 months after the diagnosis of tongue metastasis.

**Conclusions:**

Although rare, metastatic melanoma should be included in the differential diagnosis of tongue lesions detected in patients with a previous medical history of cutaneous melanoma.

** Key words:**Melanoma, tongue, metástases.

## Introduction

Melanomas are malignant tumors that originate from skin melanocytes and are considered one of the most aggressive types of cancer. Once considered a rare tumor, the incidence of cutaneous melanoma is estimated to be increasing worldwide and usually affects young adults. Solar UV radiation is a well-established risk factor for melanoma, as the most frequently affected sites include sun-exposed areas of fair-skinned patients, such as the dorsal surface of the trunk of the body, arms, legs and the head and neck region. Late diagnosis is often associated with advanced disease characterized by dissemination to regional lymph nodes and distant metastases, leading to a five-year survival rate of 5%. Surgical treatment combined with radiotherapy, chemotherapy and molecular targeted therapy have been used in the treatment of melanoma (1-3).

Primary oral melanomas account for approximately 1% of all cases; the most commonly affected intraoral sites are the palate and the maxillary gingiva. Similarly, oral metastases derived from primary melanomas located at distant sites are considered extremely rare (4). In this scenario, only eight cases of metastatic melanoma of the tongue have been reported in the English literature so far ( Table 1). Therefore, the aim of this article was to report two new cases of primary cutaneous melanoma metastasizing to the tongue and to further review the current knowledge regarding melanoma metastases to the oral cavity and tongue.

**Table 1:**
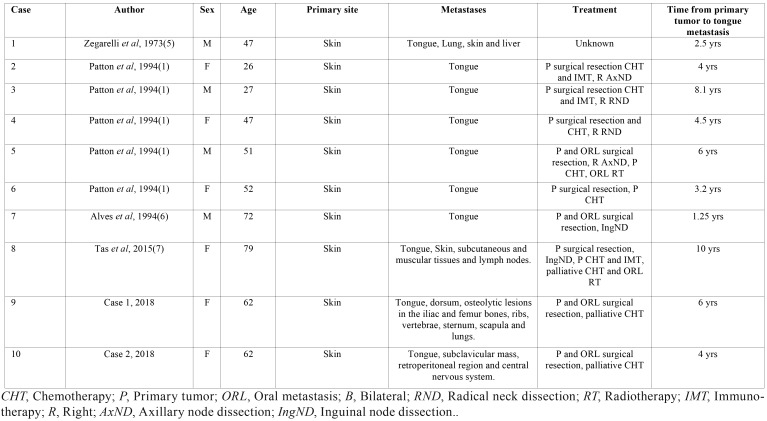
Literature review of 8 cases of melanoma metastatic to the tongue.

## Case Report

Case 1

A 62-year-old female patient was referred by her oncologist for a routine dental evaluation. The patient’s medical history revealed a cutaneous melanoma of the left dorsum diagnosed six years before she was referred to our dental service. The tumor was primarily treated by surgery and developed two local recurrences (one and three years following surgery, respectively), which were also managed by surgical resection. Disease progression was identified five years after the first treatment and confirmed by a computed tomography (CT), which revealed multiple organ involvement including the lungs, skin (subcutaneous nodules on the dorsum) and bone (osteolytic lesions in the iliac and femur bones, ribs, vertebrae, sternum and scapula).

The patient was undergoing a palliative treatment protocol based on dacarbazine, zoledronic acid and radiotherapy in the lumbar region (total dose of 20 Gy) and in the left supraclavicular fossa (total dose of 36 Gy) when she was referred to our dental facility. An extraoral clinical examination identified a pigmented subcutaneous nodule on the patient’s dorsum, measuring 5 cm in diameter, and scarring from the previous surgical resections.

**Figure 1:**
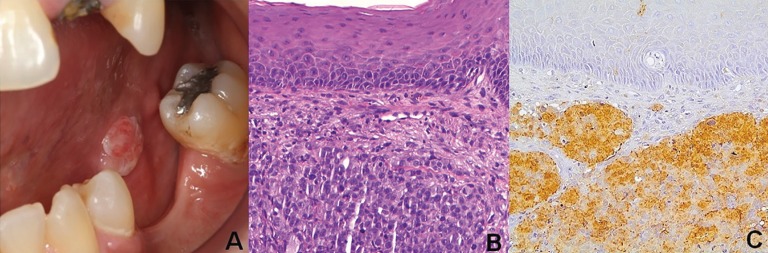
A. Clinical aspect of an exophytic nodule on the left posterior border of the tongue. B. Histopathological findings of the tongue lesion revealing the proliferation of atypical, pleomorphic, malignant tumor cells. C. Immunohistochemistry positivity for S100.

The patient’s chief complaint was the loss of the dental crown of her upper left incisor. She denied oral pain or the existence of any relevant oral soft tissue lesion. Intraoral soft tissue examination revealed a nodule with an ulcerated surface and areas of telangiectasia, on the posterior left lateral border of the tongue, measuring approximately 1 cm in diameter, adjacent to a partial edentulous mandibular area (Fig. 1A). Based on the clinical features of the tongue lesion and on the patient’s medical background, the diagnostic hypothesis included metastatic melanoma, squamous cell carcinoma and fibrous hyperplasia.

An incisional biopsy was performed under local anesthesia. The histopathological analysis showed nests and solid sheets of non-pigmented atypical cells with an epithelioid phenotype infiltrating the sub-epithelial connective tissue. A high mitotic index was observed (Fig. 1B). Tumor cells displayed diffuse immustaining for S100 protein. Other melanocytic markers such as HMB-45 and Melan-A were negative. (Fig. 1C).

After the diagnosis of a metastatic lesion on the tongue, the patient was referred back to the clinical oncologist who maintained her in a palliative protocol of chemotherapy with cisplatin, dacarbazine and vinblastine (CVD). Unfortunately, the patient died due to disease progression four and a half months after the diagnosis of tongue metastasis.

Case 2

A 62-year-old female was diagnosed with an acral lentiginous melanoma on the right heel four years previously. At that time, CT revealed pulmonary nodules and the patient was kept in close follow-up. Two years after the initial diagnosis, the patient was submitted to a pulmonary biopsy with a diagnosis of metastatic melanoma. Four months later, the patient requested a dental evaluation due to a fast-growing submucosal nodular lesion on her tongue.

**Figure 2:**
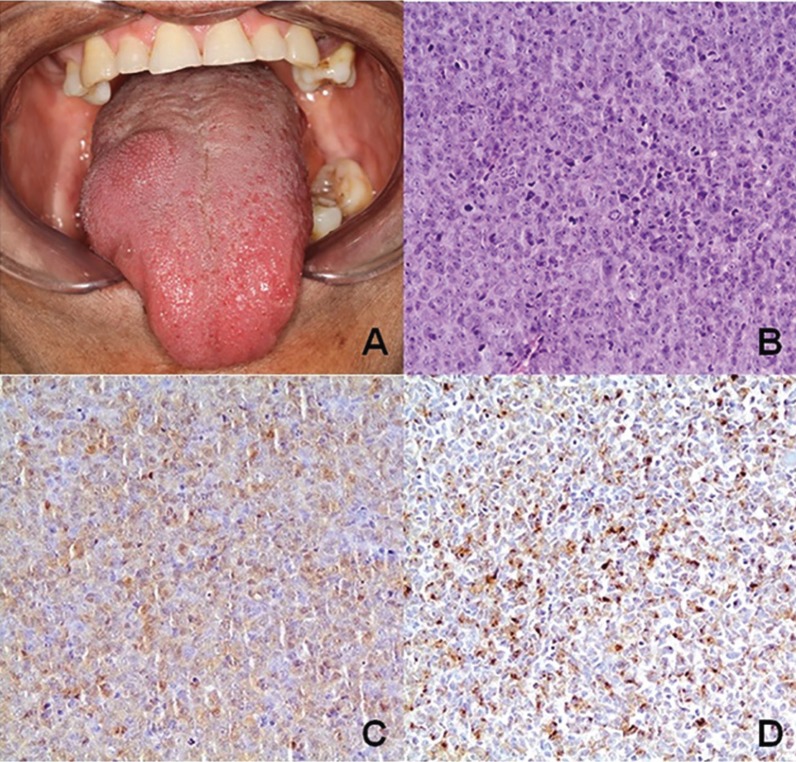
A. Submucosal nodular lesion in the tongue. B. Histopathological findings of an oral lesion showing epithelioid malignant cells. C. Immunoreactivity for HMB-45. D. and Melan-A.

During the first dental appointment, intraoral soft tissue examination revealed a submucosal nodule in the right dorsum of the tongue measuring approximately 2 cm in diameter and firm upon palpation (Fig. 2A). The patient reported three months of painless nodule progression. Based on the previous medical history of the patient, the diagnostic hypothesis included metastatic melanoma and benign mesenchymal neoplasm.

An intra-oral incisional biopsy was performed under local anaesthesia. The histopathologic analysis was characterized by proliferation of atypical epithelioid cells displaying a poorly delimited cytoplasm and hyperchromatic nucleus which contained eosinophilic macronucleoli (Fig. 2B). Immunohistochemistry showed multifocal staining for melanocytic markers, including S100 (Fig. 2C), HMB-45 (Fig. 2D) and Melan-A (Fig. 2E). These findings confirmed the clinical hypothesis of metastatic melanoma.

After diagnosis, the patient was referred back to the clinical oncologist who investigated a left subclavicular mass with a diagnosis of metastatic melanoma and also presented disease progression to the lungs, retroperitoneal region and central nervous system. The patient died 20 months after the diagnosis of oral metastatic melanoma even though she was under palliative care and careful medical follow-up.

## Discussion

The oral region is an uncommon site for metastatic tumor cell colonization. Still, there are many reported cases of oral metastases being the first indication of undiscovered malignancies. In the oral soft tissues, the attached gingiva seems to be the most common site for metastatic colonization and it usually resembles a hyperplastic or reactive lesion, such as pyogenic granuloma, peripheral giant cell granuloma, or fibrous epulis. In locations other than the gingiva, it may present as a submucosal mass(8), as seen in case 2.

Both cases of metastatic melanoma described in this report had primary tumors of the skin and one of them was a clinical finding during a dental assessment. A gap of several years between the time of treatment of primary melanoma and the appearance of metastasis as seen in these reports is a common finding(9), and metastases to the head and neck mucosa are frequent manifestations of widespread disease(10). In the present paper, patient 1 presented disease progression including subcutaneous nodules on the dorsum, osteolytic lesions in the iliac and femur bones, ribs, vertebrae, sternum, scapula and a pulmonary mass, while patient 2 presented metastatic disease in the lung, subclavicular and retroperitoneal region and central nervous system. The literature review provided eight complete detailed cases of melanoma metastatic to the tongue available in the English language (1,5,6,7) ( Table 1). Mean patient age in these cases was 50 years and cutaneous melanoma was the most frequent primary site accounting for all primary tumors. One case (12.5%) of oral metastasis was treated exclusively by surgery, one (12.5%) was treated by surgery and local adjuvant radiotherapy and one (12.5%) was treated by palliative chemotherapy and local radiotherapy. Both cases described in this report were treated first by surgical resection and after the diagnosis of distant metastases by chemotherapy with palliative purposes. The mean time for developing a tongue metastasis in the literature review was 4.9 years (ranging from 1.25 to 10 years), while in our report, one patient presented a tongue metastasis six years after the initial diagnosis and the other after four years.

A consensus is lacking on treatment for metastatic melanoma. Systemic therapy is based on many chemotherapeutic protocols; however, these have been used with varying success and little improvement in median survival. Immunomodulatory agents are also used but have low response rates, many side effects and are not recommended for all patients (11). 

 Hope relies in targeted agents such as ipilimumab and vemurafenib, which are revolutionizing the treatment of metastatic melanoma (12,13). Both patients in our report had many metastatic lesions and were treated by palliative protocols due to the inability to control disease progression. The prognosis in these cases is often very bad, and both patients died after 4 and a half and 20 months respectively.

Melanomas can histologically mimic a wide variety of neoplasms, including poorly differentiated carcinomas, high-grade lymphomas and even round cell sarcomas (6). Besides the epithelioid phenotype, the intracytoplasmic melanin deposits and eosinophilic macronucleolus are the most reliable histomorphological clues to melanoma diagnosis. However, some cases do not show the usual features and may represent a challenge for the precise microscopic detection. In the metastatic disease, it is not uncommon to see amelanotic lesions, probably due to the selection of poorly differentiated tumor cell clones. In addition, not only the pigment can be lessened or even absent but also the nuclear features and/or the cytoplasmic conformation. Spindle cell melanomas may be difficult to distinguish from round cell sarcomas, sarcomatoid carcinomas, or even an inflammatory scarring process (14). Small cell tumor phenotype includes other differential diagnoses, such as lymphoproliferative diseases and round cell sarcomas. In such settings, immunohistochemistry represent a highly valuable diagnostic tool. The most useful melanocytic-related markers are S100 protein, HMB-45 and Melan-A (also known as MART-1). All of them are expected to be positive in melanocytic neoplasms, even though their sensitivity and specificity may vary, with S100 protein being regarded as the most sensitive and HMB-45 and Melan-A as the most specific. This consistent melanocytic profile should be associated to negative immunostaining for other malignancies coming from the histomorphological analysis, such as lymphoid markers (CD45, CD20, CD3), epithelial markers (cytokeratins, epithelial membrane antigen, BER-EP4), mesenquimal markers (WT-1, CD99, desmin, muscle actins) and neuroendocrine markers (sinaptophysine, cromogranin), among others.

## Conclusion

Tongue metastases of melanoma are rare and are associated with widespread disease and consequently poor prognosis and low survival rates, as seen in the cases described here. Health professionals should always give importance to the patient’s medical history and perform a systematic oral examination to provide an early diagnosis to improve treatment and survival. Finally, metastatic tumors should always be included in the differential diagnosis of oral lesions in patients with a previous medical history of cancer.
